# Wu Shan Shen Cha (*Malus asiatica* Nakai. Leaves)-Derived Flavonoids Alleviate Alcohol-Induced Gastric Injury in Mice via an Anti-Oxidative Mechanism

**DOI:** 10.3390/biom9050169

**Published:** 2019-05-03

**Authors:** Bihui Liu, Chengfeng Zhang, Jing Zhang, Xin Zhao

**Affiliations:** 1 Chongqing Collaborative Innovation Center for Functional Food, Chongqing University of Education, Chongqing 400067, China; liubh@foods.ac.cn; 2 Chongqing Engineering Research Center of Functional Food, Chongqing University of Education, Chongqing 400067, China; 3 Chongqing Engineering Laboratory for Research and Development of Functional Food, Chongqing University of Education, Chongqing 400067, China; 4 Biological and Chemical Engineering Collage, Chongqing University of Education, Chongqing 400067, China; zhangcf@foods.ac.cn; 5 Environment and Quality Inspection College, Chongqing Chemical Industry Vocational College, Chongqing 401228, China; zhangjing@foods.ac.cn

**Keywords:** *Malus asiatica* Nakai., flavonoid, alcoholic gastric injury, mRNA expression, oxidation

## Abstract

Wu Shan Shen Cha is the leaf of *Malus asiatica* Nakai., a special type of tea that is consumed in the same way as green tea. To study the effect of Wu Shan Shen Cha-derived flavonoids (WSSCF) on lesions in the stomach, a 15% hydrochloric acid–95% ethanol (volume ratio 4:6) solution was used to induce gastric injury in mice. The degree of gastric injury was assessed using tissue specimens, and the effects of WSSCF on the serum levels of antioxidant enzymes were investigated. The results showed that WSSCF could alleviate the damage of the gastric mucosa and gastric wall caused by the hydrochloric acid–ethanol solution, decrease the tissue and serum levels of malondialdehyde (MDA) in mice with gastric injury, and increase the serum levels of superoxide dismutase (SOD) and glutathione (GSH). The results of quantitative polymerase chain reaction (qPCR) showed that WSSCF could increase the mRNA expression of Mn-SOD, Cu/Zn-SOD, catalase (CAT), endothelial nitric oxide synthase (eNOS), and neuronal nitric oxide synthase (nNOS) in tissue specimens from mice with gastric injury and decrease the expression of cyclooxygenase-2 (COX-2) and inducible nitric oxide synthase (iNOS). At the same time, the results of the high concentration of WSSCF (WSSCFH) group were closer to those of the drug (ranitidine) treatment group. Wu Shan Shen Cha-derived flavonoids had a good antioxidant effect, so as to play a preventive role in alcoholic gastric injury.

## 1. Introduction

Alcohol (ethanol) consumption by humans is on the rise, and the incidence of digestive tract diseases in the drinking population is significantly higher than that in the non-drinking population [1]. In addition to the liver, the gastrointestinal tract also participates in the metabolism of ethanol, and the process requires various enzymes, including ethanol dehydrogenases (ADH), cytochrome P450, and catalase [2]. Free radicals are also involved in alcohol-induced gastric mucosal injury [3]. The levels of lipid peroxides and free radicals in the gastric mucosa of alcoholic drinkers were elevated. Oxygen free radicals acting on mitochondria can reduce ATP production, cause Na^+^-K^+^-ATPase dysfunction, increase the intracellular Na^+^ concentration, trigger Na^+^-Ca^2+^ exchange, and increase intracellular calcium. A high calcium concentration can activate phospholipase A2, promote phospholipase degradation, decrease the membrane phospholipid content, and increase the membrane permeability to Ca^2+^ [3]. In addition, reactive oxygen species can directly inhibit Ca^2+^-Mg^2+^-ATPase, suggesting that oxygen free radicals may trigger calcium overload [4]. The damage of the gastric mucosa caused by ethanol may be related to the decreased fluidity of cell membranes caused by excessive oxygen free radicals and intracellular calcium overload [5].

Wu Shan Shen Cha, produced in the Three Gorges Valley of the Yangtze River in China, is a type of tea refined from the leaves of the unique and local *Malus asiatica* Nakai. This tea comes from a pure wild plant that is rich in various trace elements, polyphenols, and flavonoids, which are beneficial to human health. It is a natural beverage for relieving thirst, reducing fatigue and weight, lowering blood pressure, awakening the brain, and providing spiritual nourishment [6]. The local people refer to Wu Shan Shen Cha as a longevity tea, and it appears to have antioxidant effects. Flavonoids are one of the most important components of this tea. According to previous studies, flavonoids are potent compounds with bacteriostatic, anti-free radical, anti-oxidative, insecticidal, anti-cancer, anti-aging, and lipid- and cholesterol-lowering effects [7,8,9]. Flavonoids are strong antioxidants, which can scavenge oxygen free radicals in the body, decrease the lipid peroxide level, and prevent oxidation ten times more effectively than vitamin E. These anti-oxidative effects can prevent cell degeneration, aging, and other conditions [10]. In addition, flavonoids can trigger the activities of antioxidant enzymes, such as superoxide dismutase (SOD), glutathione (GSH), and catalase (CAT), thus effectively scavenging free radicals, and it can also inhibit malondialdehyde (MDA)-induced lipid peroxidation and prevent cell damage [11]. Recent studies have shown that most of the physiological activities of plant flavonoids are based on their anti-oxidant effects [12,13]. In recent years, several studies have reported that tea exacerbates alcohol-induced gastric injury, but there is no in-depth study on the effects of Wu Shan Shen Cha on alcohol-induced gastric injury, possibly due to its limited consumption.

In this study, the effects of flavonoids extracted from Wu Shan Shen Cha (WSSCF) on alcohol-induced gastric injury were studied in mice, and the mechanism of action was defined. It was found that WSSCF mediated their biological effects through an anti-oxidative mechanism, which provides a theoretical basis for the application of Wu Shan Shen Cha as a traditional resource.

## 2. Materials and Methods

### 2.1. Wu Shan Shen Cha-Derived Flavonoids Extraction

Approximately 100 g of WSSC was crushed into powder, and 60 g of WSSC powder, 1.3 g of Na_2_CO_3_, and 2.6 g of Na_2_B_4_O_7_ were added to 600 mL of a 40% (volume ratio) ethanol solution. WSSC was soaked in water at 50 °C for 30 min and centrifuged (4000 rpm, 10 min), followed by the collection of the supernatant. Flavonoids in the precipitate were extracted using the aforementioned procedure, and the supernatants from two centrifugation steps were combined. The pH was adjusted to 4.0 with hydrochloric acid, and the flavonoid extract was obtained using steam-drying ethanol rotary evaporation.

### 2.2. Determination of the Flavonoid Extract Purity

Rutin was dissolved in 90% ethanol solution to dilute the compound to concentrations of 10, 20, 30, 40, and 50 μg/mL. The Kudingcha flavonoid extract was also dissolved in a 90% ethanol solution. According to the standard rutin solution curve, the absorbance was determined at 500 nm with a spectrophotometer, and the purity of the flavonoid extract in the WSSCF extract was calculated.

### 2.3. Establishment of the Animal Model

Specific pathogen-free six-week-old male Kunming mice were randomly divided into normal, model, low WSSCF concentration (WSSCFL, 100 mg/kg), high WSSCF concentration (WSSCFH, 200 mg/kg), and ranitidine (50 mg/kg) groups with 10 mice in each group. After feeding for 7 days, the mice in the normal and model groups were administered 2 mL of distilled water daily for 14 days, the mice in the WSSCFL group were administered 0.2 mL of WSSCF (100 mg/kg) daily for 14 days, the mice in the WSSCFH group were administered 0.2 mL of WSSCF (200 mg/kg) daily for 14 days, and the mice in the ranitidine group were administered ranitidine (50 mg/kg) daily for 14 days. After treating for 14 days, the mice in all groups were fasted but were allowed to freely drink water. After fasting for 1 day (i.e., on the 15th day), the mice in all groups, except those in the normal group, were administered 15% hydrochloric acid–95% ethanol (volume ratio 4:6) at 0.1 mL/10 g body weight. After treating for 30 min, the mice were euthanized. Plasma was collected from the eyeballs, and stomach specimens were harvested [13]. The volume of gastric juice, the secretion of gastric juice, and the pH of the gastric acid were determined. After washing the stomach specimens, the degree of gastric injury was assessed as follows:

Inhibitory rate of gastric injury (%) = area of gastric injury/area of gastric tissue.

This study was conducted in accordance with the Declaration of Helsinki, and the protocol was approved by the Ethics Committee of Chongqing Collaborative Innovation Center for Functional Food (201807003B).

### 2.4. Pathological Examination of Gastric Specimens from Mice

Gastric specimens were fixed in 10% formalin for 48 h, followed by dehydration, clearing, embedment in wax, and sectioning. The sections were stained with hematoxylin and eosin and observed under a BX43 optical microscope (Olympus, Tokyo, Japan).

### 2.5. Determination of Total Superoxide Dismutase, Glutathione Activities and Malondialdehyde Content in Serum and Gastric Tissues in Mice

Nitrite is the final product of oxidizing hydroxyl group of ·O_2_. Nitrite shows purple-red under the action of p-aminobenzene sulfonic acid and methylnaphthalene amine. It has a maximum absorption peak at 550 nm. Total superoxide dismutase (T-SOD) can be calculated by determining the nitrite reduced after SOD scavenging ·O_2_. GSH in the sample reacts with 5,5-dithio-bis-(2-nitrobenzoic acid) (DTNB) to produce stable yellow 5-thio-2-nitrobenzoic acid (TNB) and glutathione disulfide (GSSG). The GSH content of the sample can be calculated. Under acidic and high-temperature conditions, MDA can react with thiobarbituric acid (TBA) to form red-brown trimethadione; its maximum absorption wavelength is 532 nm. Whole blood was collected from mice, stored for 1 h, and centrifuged at 4000 rpm for 10 min. The serum was collected. The serum and tissue levels of T-SOD, GSH, and MDA were determined according to the manufacturer’s instructions (Nanjing Jiancheng Bioengineering Institute, Nanjing, China).

### 2.6. Quantitative Polymerase Chain Reaction Assay

Stomach specimens were homogenized. Total RNA was extracted using RNAzol (Thermo Fisher Scientific, Waltham, MA, USA), and the concentration was diluted to 1 μg/μL. Thereafter, 5 μL RNA was reverse transcribed to prepare DNA. Approximately 2 μL of DNA was combined with 1 μL of the forward primer, 1 μL of the reverse primer (Table 1), and 10 μL of SYBR Green PCR Master Mix and amplified at 95 °C for 60 s, followed by 40 cycles at 95 °C for 15 s, 55 °C for 30 s, and 72 °C for 35 s. Glyceraldehyde-3-phosphate dehydrogenase (GAPDH) served as the internal control. The relative expression of each target gene was calculated by the 2^−ΔΔCt^ method [13].

### 2.7. Statistical Analysis

All experiments were performed three independent times, and the data were averaged. Statistical analysis system (SAS) 9.1 Statistical Software (SAS Institute, Cary, NC, USA) was used for statistical analyses. One-way analysis of variance was used to test differences between the means of the groups. *p*-Values < 0.05 were considered significant.

## 3. Results

### 3.1. Content of Wu Shan Shen Cha-Derived Flavonoids

**Figure 1:**
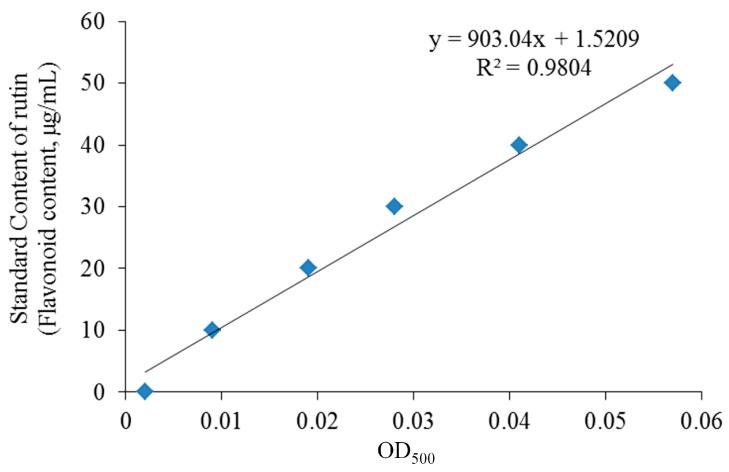
The standard curve of flavonoid content (rutin).

By observing the absorbance value of rutin standard at different concentrations, the standard curve (*y* = 903.04*x* + 1.5209, *R*^2^ = 0.9804, Figure 1) of the relationship between the concentration of rutin and the absorbance value was established. By measuring the absorbance value of WSSCF, compared with the standard curve, the content of flavonoids in the WSSCF reached 82.7%, and the purity was high. In the subsequent animal experiments, the flavonoids in the WSSCF were the main active ingredients and played a central role.

### 3.2. Determination of Gastric Injury in Mice

**Figure 2:**
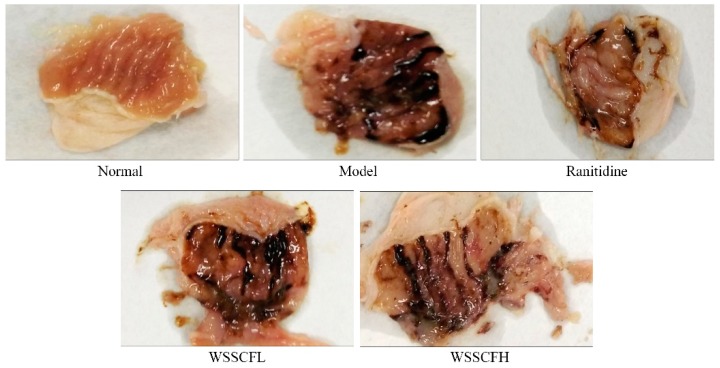
Images of stomach specimens from mice of each group. Ranitidine at 50 mg/kg body weight (b.w.) by gavage, Wu Shan Shen Cha flavonoids at 100 mg/kg b.w. by gavage for the low concentration of Wu Shan Shen Cha-derived flavonoids (WSSCFL) group; and Wu Shan Shen Cha flavonoids at 200 mg/kg b.w. by gavage for the high concentration of Wu Shan Shen Cha-derived flavonoids (WSSCFH) group.

Figure 2 and Table 2 show that the gastric lesions in mice of the model group were the most severe. Compared to gastric lesions in the mice of the model group, those in the mice of the WSSCF groups (i.e., low and high concentrations) were less severe. The gastric lesions in mice of the ranitidine group were the least severe, and the inhibition rate of gastric injury was the highest. These results indicate that lesions in mice of the WSSCFH group were similar to those in mice of the ranitidine group, suggesting that WSSCF can alleviate alcohol-induced gastric injury.

### 3.3. Determination of the Volume and pH of the Gastric Fluid in Mice

In mice of the normal group, the gastric juice volume was the lowest, and the gastric juice pH was the highest (Table 3). In mice of the model group, the gastric juice volume was the highest and the gastric juice pH was the lowest. WSSCF and ranitidine effectively decreased the gastric juice volume and increased the gastric juice pH of mice in the model group, and the effects of WSSCFH were similar to those of ranitidine.

### 3.4. Histopathological Examination of the Stomach in Mice

**Figure 3:**
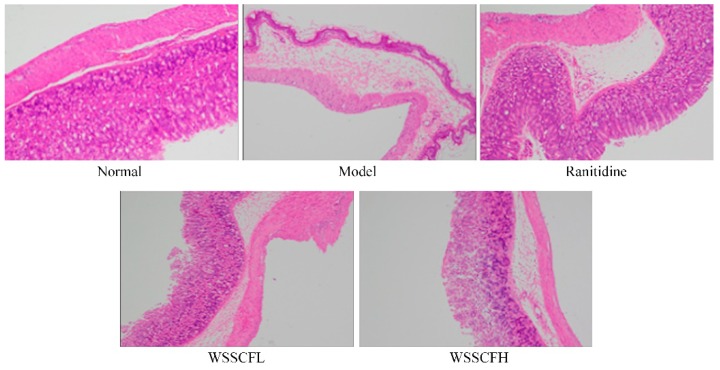
Images of hematoxylin and eosin-stained stomach sections of mice in each group (100×). Ranitidine at 50 mg/kg b.w. by gavage; Wu Shan Shen Cha flavonoids at 100 mg/kg b.w. by gavage for the WSSCFL group; and Wu Shan Shen Cha flavonoids at 200 mg/kg b.w. by gavage for the WSSCFH group are shown.

Stomach sections of mice in each group were stained with hematoxylin and eosin and examined under a light microscope. As shown in Figure 3, the gastric mucosa of mice in the normal group was intact, and no inflammatory cell infiltration was observed. The gastric mucosa of mice in the model group was damaged. We observed a large number of red blood cells and neutrophils infiltrating the lamina propria. The gastric mucosa of WSSCFL-treated mice was similar to that of untreated mice (i.e., the normal group), and the gastric mucosa of WSSCFH-treated mice was similar to that of untreated mice (i.e., the model group). Specifically, there was less tissue damage (i.e., improved gastric injury) and fewer neutrophils and red blood cells. The gastric mucosa of mice in the ranitidine group was similar to that of mice in the normal group, with very little damage.

### 3.5. Measurement of Total Superoxide Dismutase, Glutathione and Malondialdehyde Levels in the Serum and Stomach Tissues of Mice

The levels of T-SOD and GSH in mice of the normal group were the highest (Table 4 and Table 5), whereas the MDA level was the lowest. After inducing gastric injury, the levels of T-SOD and GSH in mice of the model group decreased, whereas the MDA increased. Ranitidine and WSSCF effectively inhibited the decrease in the levels of T-SOD and GSH as well as the increase in the MDA level in mice with gastric injury, and the effects of WSSCFH were similar to those of ranitidine.

### 3.6. Cu/Zn-Superoxide Dismutase, Mn-Superoxide Dismutase, and Catalase mRNA Expression in Stomach Tissues of Mice

**Figure 4:**
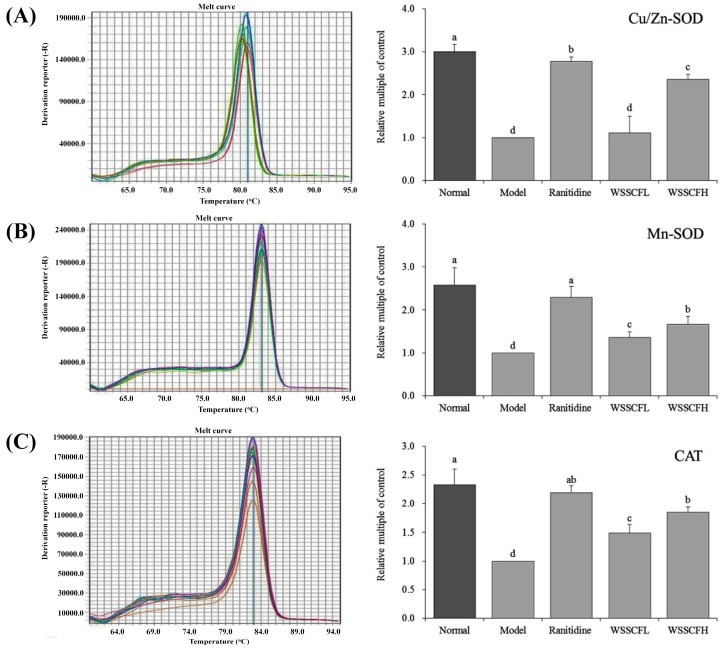
mRNA expression of Cu/Zn-SOD (**A**), Mn-SOD (**B**), and CAT (**C**) in stomach tissues from mice of each group. Values presented are the means ± standard deviation. ^a–d^ Mean values with different letters in the same bars are significantly different (*p* < 0.05) according to Duncan’s multiple-range test. Ranitidine at 50 mg/kg b.w. by gavage; Wu Shan Shen Cha flavonoids at 100 mg/kg b.w. by gavage for the WSSCFL group; and Wu Shan Shen Cha flavonoids at 200 mg/kg b.w. by gavage for the WSSCFH group are shown.

The expression of Cu/Zn-SOD, Mn-SOD, and CAT in stomach tissues of mice is shown in Figure 4. The mRNA levels of Cu/Zn-SOD, Mn-SOD, and CAT were significantly higher (*p* < 0.05) in mice of the normal group than those in mice of the other groups. The mRNA levels of Cu/Zn-SOD, Mn-SOD, and CAT were higher in mice of the WSSCF and ranitidine groups than in mice of the control group. The mRNA levels of Cu/Zn-SOD, Mn-SOD, and CAT were similar between mice of the WSSCFH and ranitidine groups.

### 3.7. Cyclooxigenase-2, eNOS, nNOS and iNOS mRNA Expression in Stomach Tissues of Mice

**Figure 5:**
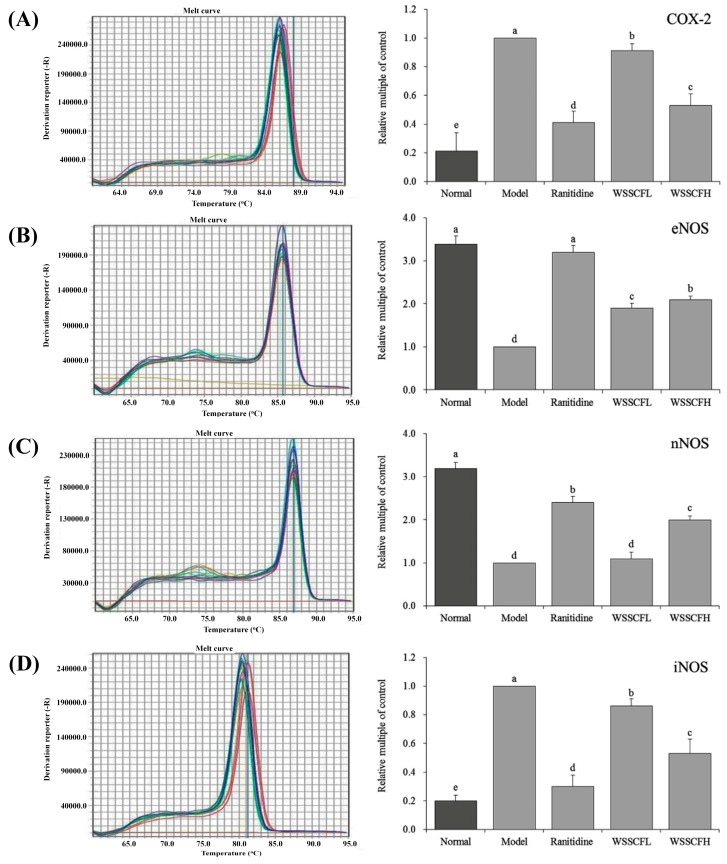
mRNA expression of COX-2 (**A**), eNOS (**B**), nNOS (**C**), and iNOS (**D**) in stomach tissues from mice of each group. Values presented are the means ± standard deviation. ^a–e^ Mean values with different letters in the same bars are significantly different (*p* < 0.05) according to Duncan’s multiple-range test. Ranitidine at 50 mg/kg b.w. by gavage; Wu Shan Shen Cha flavonoids at 100 mg/kg b.w. by gavage for the WSSCFL group; and Wu Shan Shen Cha flavonoids at 200 mg/kg b.w. by gavage for the WSSCFH group are shown.

The expression of cyclooxygenase (COX)-2, iNOS, eNOS, and nNOS in stomach tissues of mice is shown in Figure 5. The mRNA levels of the inflammatory factors COX-2 and iNOS were significantly lower (*p* < 0.05) in mice of the WSSCF group than those in mice of the model group, but higher than those in normal and ranitidine groups. By contrast, the mRNA levels of eNOS and nNOS were significantly higher (*p* < 0.05) in mice of the WSSCF group than those in mice of the model group, but lower than those in the normal group. The mRNA levels of COX-2, iNOS, eNOS, and nNOS were similar among mice of normal, WSSCFH, and ranitidine groups.

## 4. Discussion

It is generally recommended that the human body drink 12 g of tea brewed every day. According to the content of 5% flavonoids, the human body intakes 600 mg of flavonoids per day and 10 mg/kg of tea flavonoids per day. The intake of flavonoids in transformed mice was about 100 mg/kg, so we chose 100 and 200 mg/kg as the experimental concentration. Flavonoids are well absorbed by the digestive system. In addition to being mainly absorbed in the large intestine, they can also be absorbed in the stomach and small intestine and play a better role through liver metabolism. The intragastric concentration used in this study was calculated according to the recommended drinking concentration of human body, so flavonoids might have good absorption and utilization rates in mice and would not produce metabolic accumulation.

Alcohol produces acetaldehyde via the catalysis of ethanol dehydrogenase, and acetaldehyde produces oxygen free radicals via the action of xanthine oxidase, resulting in gastric mucosal damage. Free radical reactions are closely related to the occurrence and development of various gastrointestinal conditions [14]. According to a previous study, the annual per capita alcohol intake exceeds 10 L, and the alcohol intake of males is close to 15 L [15]. Studies have reported that the incidence of gastrointestinal diseases in the drinking population is significantly higher than that in the non-drinking population. The frequent intake of high-concentration alcohol can cause thinning of the gastric mucosa, necrosis, and exfoliation of epithelial cells, microvascular endothelial injury, embolism, tissue ischemia, and hypoxia necrosis, so frequent intake of alcohol can cause gastric mucosal erosion, ulcer formation, gastroduodenal mucosal injury, and other related gastric diseases [16,17]. The abundant and bioactive substances in tea, especially flavonoids, benefit the microbial community in the intestinal tract and inhibit intestinal mucosal atrophy. The anti-oxidative properties of flavonoids also protect the intestinal mucosal barrier and the liver [18].

Lesions can alter the composition of gastric juice due to the excessive formation of free radicals, and secretion of gastric juice or low gastric juice pH will aggravate the degree of gastric injury [19]. The results of this study showed that WSSCF reduced the volume of gastric juice secretion without altering its pH, thereby alleviating the gastric injury caused by the high concentration of ethanol.

Several studies have reported a correlation between alcohol-induced gastric injury and high levels of reactive oxygen species [20,21]. There are enzymatic and non-enzymatic antioxidant enzymes in the body, including SOD, GSH, and CAT. Studies have shown that GSH and SOD can scavenge superoxide, hydrogen peroxide, hydroxyl, and lipid peroxide free radicals, thereby reducing oxidative damage to tissues [19]. MDA is a marker of lipid peroxidation, and its content is positively correlated with the degree of cell damage [22]. When reactive oxygen species exceed the scavenging capacity of the antioxidant enzymes, the gastrointestinal tract will be severely damaged, resulting in tissue damage [23]. In this study, WSSCF increased the levels of SOD and GSH in mice, whereas it decreased the MDA level, thereby protecting the gastric tissue from oxidative damage caused by ethanol.

Copper/Zinc-SOD and Mn-SOD are two isomers of SOD in mammals, and both are free radical scavengers of SOD, except that they contain different metal ions. Cu/Zn-SOD mainly exists in the cytoplasm, whereas Mn-SOD mainly exists in the mitochondria and prokaryotic cells. Mn-SOD is abundant in visceral tissues [24]. Among them, Mn-SOD catalyzes the disproportionation of superoxide anion radicals into hydrogen peroxide, which can be decomposed into hydrogen peroxide to achieve the balance of intracellular physiological functions. It is of great significance to maintain the normal state of cell redox reaction [25,26]. The activity of Mn-SOD decreased significantly after alcohol-induced gastric injury induced in mice. Cu/Zn-SOD can reduce the toxic effect of O_2_^−^ and protect gastric tissue. Studies have shown that alcohol causes oxidative damage and produces various free radicals. Mn-SOD and Cu/Zn-SOD can inhibit free radicals in vivo and prevent gastric injury [27]. Catalase (CAT) is an enzymatic scavenger and an important antioxidant enzyme. It can promote the decomposition of hydrogen peroxide into molecular oxygen and water, thereby eliminating hydrogen peroxide, protecting cells from hydrogen peroxide poisoning, inhibiting oxidative damage, reducing the oxidation caused by alcohol, and inhibiting gastric injury [28].

Megatrophils synthesize and release nitric oxide (NO). Excessive NO can inhibit energy production, ribonucleoside reductase activity, and DNA replication. In particular, excessive NO production can induce cytotoxic effects, DNA oxidation, DNA double-strand breakage, cross-linking, and base modification. In gastric mucosa, NO functions as a signal transducer between immune cells and epithelial cells. It not only protects the gastric mucosa, but also promotes inflammation to damage the gastric mucosa [29]. The reactive nitrogen species (RNS) oxidative pathway is related to nitric oxide. After gastric tissue undergoes oxidative damage, the balance is broken, and gastric tissue damage is aggravated [30]. NOS is the key enzyme for NO synthesis, and nitric oxide synthase in the gastric mucosa can be divided into two types: cNOS, including eNOS and nNOS, and iNOS. Low levels of NO produced by cNOS catalysis can protect the gastric mucosa, whereas gastric ulcers are caused by the excessive inhibition of eNOS and nNOS [30]. Cyclooxygenase is a kind of key enzyme associated with inflammation. Under normal conditions, COX-2 is weakly expressed, but cytokines and endotoxins can induce its production, and COX-2 can induce inflammation and promote inflammation with oxidative stress, aggravating gastric injury [31].

At present, the use of Wu Shan Shen Cha is not enough just as a tea drink. WSSCF is a kind of important active substance in Wu Shan Shen Cha. It can be made into a single functional food or used as a high-quality functional food additive to exert its biological activity on the human body. However, its industrial extraction technology and processing technology in functional food need further study.

## 5. Conclusions

In this study, the protective effects of flavonoid extracted from Wu Shan Shen Cha (WSSCF) on alcohol-induced gastric injury in mice were evaluated. It was found that WSSCF could inhibit the secretion of gastric juice in mice with gastric injury and maintain the normal pH of gastric acid, thus alleviating the damage of the gastric mucosa. By hematoxylin and eosin staining, the degree of gastric mucosal damage in WSSCF-treated mice was alleviated compared with that in model mice. WSSCF increase the serum levels of SOD and GSH, reduced the MDA content, and protected the gastric mucosa from oxidative damage caused by free oxygen radicals, which indicates that WSSCF has good antioxidant effect in vivo. Further experiments showed that WSSCF increased the mRNA expression of eNOS and nNOS and decreased the expression of COX-2 in gastric tissues, revealing that WSSCF can exert its biological effects by functioning as an antioxidant agent. Moreover, with increasing WSSCF in the stomach, the alcohol-induced gastric injury in mice was alleviated. This shows that WSSCF has better health care effect and provides a theoretical basis for its further development and utilization.

## Figures and Tables

**Table 1:** Sequences of the primers used in qPCR assays.

Gene	Sequence
*Cu/Zn-SOD*	Forward: 5′–AACCAGTTGTGTTGTCAGGAC–3′
	Reverse: 5′–CCACCATGTTTCTTAGAGTGAGG–3′
*Mn-SOD*	Forward: 5′–CAGACCTGCCTTACGACTATGG–3′
	Reverse: 5′–CTCGGTGGCGTTGAGATTGTT–3′
*CAT*	Forward: 5′–GGAGGCGGGAACCCAATAG–3′
	Reverse: 5′–GTGTGCCATCTCGTCAGTGAA–3′
*COX-2*	Forward: 5′–GGTGCCTGGTCTGATGATG–3′
	Reverse: 5′–TGCTGGTTTGGAATAGTTGCT–3′
*nNOS*	Forward: 5′–GAGAGGATTCTGAAGATGAGG–3′
	Reverse: 5′–TTGCTAATGAGGGAGTTGTTC–3′
*eNOS*	Forward: 5′–TGTTTGTCTGCGGCGATGT–3′
	Reverse: 5′–GGGTGCGTATGCGGCTTGTC–3′
*iNOS*	Forward: 5′–CATTGGAAGTGAAGCGTTTCG–3′
	Reverse: 5′–CACAGAACTGAGGGTACA–3′
*GAPDH*	Forward: 5′–AGGTCGGTGTGAACGGATTTG–3′
	Reverse: 5′–GGGGTCGTTGATGGCAACA–3′

qPCR: Quantitative polymerase chain reaction; *Cu/Zn-SOD*: Copper/zinc-superoxide dismutase *Mn-SOD*: Manganese-superoxide dismutase; *COX-2*: Cyclooxygenase-2; *nNOS*: Neuronal nitric oxide synthase; *eNOS*: Endothelial nitric oxide synthase; *iNOS*: Inducible nitric oxide synthase; *GAPDH*: Glyceraldehyde-3-phosphate dehydrogenase.

**Table 2:** Degrees of gastric injury in mice of each group (*n* = 10).

Group	Area of Gastric Injury (mm^2^)	Inhibitory Rate of Gastric Injury (%)
Normal	0.00 ± 0.00 ^e^	100 ± 0.00 ^c^
Model	16.57 ± 0.96 ^a^	0.00 ± 0.00 ^a^
Ranitidine	5.00 ± 0.82 ^d^	69.82 ± 1.64 ^b^
WSSCFL	11.57 ± 1.93 ^b^	30.21 ± 1.67 ^a^
WSSCFH	8.57 ± 1.30 ^c^	48.26 ± 1.51 ^b^

Values presented are the means ± standard deviation. ^a–e^ Mean values with different letters in the same column are significantly different (*p* < 0.05) according to Duncan’s multiple-range test. Ranitidine at 50 mg/kg b.w. by gavage; Wu Shan Shen Cha flavonoids at 100 mg/kg b.w. by gavage for the low concentration of Wu Shan Shen Cha-derived flavonoids (WSSCFL) group; and Wu Shan Shen Cha flavonoids at 200 mg/kg b.w. by gavage for the high concentration of Wu Shan Shen Cha-derived flavonoids (WSSCFH) group are shown.

**Table 3:** Volume and pH of gastric fluid in mice of each group (*n* = 10).

Group	Gastric Juice Volume (mL)	Gastric Juice pH
Normal	0.03 ± 0.01 ^b^	3.80 ± 0.45 ^a^
Model	0.28 ± 0.09 ^a^	1.67 ± 0.58 ^b^
Ranitidine	0.19 ± 0.06 ^a,b^	1.80 ± 0.45 ^a,b^
WSSCFL	0.21 ± 0.15 ^a,b^	1.75 ± 0.50 ^a,b^
WSSCFH	0.20 ± 0.06 ^a,b^	1.80 ± 0.45 ^a,b^

Values presented are the means ± standard deviation. ^a,b^ Mean values with different letters in the same column are significantly different (*p* < 0.05) according to Duncan’s multiple-range test. Ranitidine at 50 mg/kg b.w. by gavage; Wu Shan Shen Cha flavonoids at 100 mg/kg b.w. by gavage for the WSSCFL group; and Wu Shan Shen Cha flavonoids at 200 mg/kg b.w. by gavage for the WSSCFH group.

**Table 4:** Serum levels of superoxide dismutase (T-SOD), glutathione (GSH) and malondialdehyde (MDA) in mice of each group (*n* = 10).

Group	T-SOD (U/mL)	GSH (mg/L)	MDA (nmol/mL)
Normal	234.93 ± 10.24 ^a^	10.10 ± 1.25 ^a^	14.44 ± 1.78 ^e^
Model	189.52 ± 34.80 ^e^	7.91 ± 0.47 ^e^	33.64 ± 8.15 ^a^
Ranitidine	222.08 ± 17.53 ^b^	9.55 ± 0.24 ^b^	15.05 ± 3.95 ^d^
WSSCFL	198.02 ± 22.71 ^d^	8.60 ± 1.48 ^d^	17.12 ± 4.91 ^b^
WSSCFH	218.21 ± 23.63 ^c^	9.14 ± 3.10 ^c^	15.56 ± 4.11 ^c^

Values presented are the means ± standard deviation. ^a–e^ Mean values with different letters in the same column are significantly different (*p* < 0.05) according to Duncan’s multiple-range test. Ranitidine at 50 mg/kg b.w. by gavage; Wu Shan Shen Cha flavonoids at 100 mg/kg b.w. by gavage for the WSSCFL group; and Wu Shan Shen Cha flavonoids at 200 mg/kg b.w. by gavage for the WSSCFH group are shown.

**Table 5:** Stomach tissue levels of T-SOD, GSH, and MDA in mice of each group (*n* = 10).

Group	T-SOD (U/mg protein)	GSH (mg/g protein)	MDA (nmol/mg protein)
Normal	8.38 ± 0.15 ^a^	4.15 ± 0.58 ^a^	0.54 ± 0.06 ^b^
Model	4.95 ± 0.55 ^e^	3.55 ± 1.09 ^b^	1.03 ± 0.23 ^a^
Ranitidine	7.17 ± 0.35 ^b^	3.87 ± 1.51 ^ab^	0.63 ± 0.13 ^ab^
WSSCFL	5.28 ± 0.03 ^d^	3.74 ± 0.19 ^ab^	0.71 ± 0.29 ^ab^
WSSCFH	6.45 ± 0.63 ^c^	3.85 ± 0.96 ^ab^	0.68 ± 0.22 ^ab^

Values presented are the means ± standard deviation. ^a–e^ Mean values with different letters in the same column are significantly different (*p* < 0.05) according to Duncan’s multiple-range test. Ranitidine at 50 mg/kg b.w. by gavage; Wu Shan Shen Cha flavonoids at 100 mg/kg b.w. by gavage for the WSSCFL group; and Wu Shan Shen Cha flavonoids at 200 mg/kg b.w. by gavage for the WSSCFH group are shown.

## References

[1] Grønbaek M., Becker U., Johansen D., Tønnesen H., Jensen G., Sørensen T.I. (1998). Population based cohort study of the association between alcohol intake and cancer of the upper digestive tract. BMJ.

[2] Balusikova K., Kovář J. (2013). Alcohol dehydrogenase and cytochrome P450 2E1 can be induced by long-term exposure to ethanol in cultured liver HEP-G2 cells. Vitr. Cell. Dev. Boil..

[3] Salim A.S. (1991). Protection against stress-induced acute gastric mucosal injury by free radical scavengers. Intensiv. Care Med..

[4] Bafna P., Balaraman R. (2005). Anti-ulcer and anti-oxidant activity of Pepticare, a herbomineral formulation. Phytomedicine.

[5] Zima T., Fialová L., Mestek O., Janebová M., Crkovská J., Malbohan I., Stípek S., Mikulíková L., Popov P. (2001). Oxidative stress, metabolism of ethanol and alcohol-related diseases. J. Biomed. Sci..

[6] Zhou L., Zhao X., Li G., Fu L. (2014). In vitro antioxidant and antimutagenic effects of Wu Shan Shen Cha aqueous extract. J. Changshu Inst. Technol..

[7] O’Reilly J.D., Mallet A.I., McAnlis G.T., Young I.S., Halliwell B., Ab Sanders T., Wiseman H. (2001). Consumption of flavonoids in onions and black tea: Lack of effect on F2-isoprostanes and autoantibodies to oxidized LDL in healthy humans. Am. J. Clin. Nutr..

[8] Yoshida H., Ishikawa T., Hosoai H., Suzukawa M., Ayaori M., Hisada T., Sawada S., Yonemura A., Higashi K., Ito T. (1999). Inhibitory effect of tea flavonoids on the ability of cells to oxidize low density lipoprotein. Biochem. Pharmacol..

[9] Kasaoka S., Kiriyama S., Hase K., Morita T. (2002). Green tea flavonoids inhibit the LDL oxidation in osteogenic disordered rats fed a marginal ascorbic acid in diet. J. Nutr. Biochem..

[10] Kähkönen M.P., Heinonen M. (2003). Antioxidant Activity of Anthocyanins and Their Aglycons. J. Agric. Chem..

[11] Zhang W., Dong Z., Chang X., Zhang C., Rong G., Gao X., Zeng Z., Wang C., Chen Y., Rong Y. (2018). Protective effect of the total flavonoids from *Apocynum venetum* L. on carbon tetrachloride-induced hepatotoxicity in vitro and in vivo. J. Physiol. Biochem..

[12] Huang A. (2007). Advances in research of pharmacological action of flavonoids. Auhui Agri. Sci. Bull..

[13] Qian Y., Zhang J., Fu X., Yi R., Sun P., Zou M., Long X., Zhao X. (2018). Preventive effect of raw Liubao tea polyphenols on mouse gastric injuries induced by HCl/ethanol via anti-oxidative stress. Molecules.

[14] Mota C.S., Freitas R.B., Athayde M.L., Boligon A.A., Augusti P.R., Somacal S., Rocha M.P., Bauermann L.F. (2011). Effect of *Vernonia cognata* on oxidative damage induced by ethanol in rats. Hum. Exp. Toxicol..

[15] Li X.J., Tang W., Xu H., Jiang M.D., Zhou J., Mo B., He Q.W. (2013). Clinical analysis of alcoholic liver cirrhosis in 62 cases. Med. J. Nat. Defend. For. Southwest China.

[16] Taylor B., Rehm J. (2005). Moderate Alcohol Consumption and Diseases of the Gastrointestinal System: A Review of Pathophysiological Processes. Dig. Dis..

[17] Mathews S., Xu M., Wang H., Bertola A., Gao B. (2014). Animals Models of Gastrointestinal and Liver Diseases. Animal models of alcohol-induced liver disease: Pathophysiology, translational relevance, and challenges. Am. J. Physiol. Liver Physiol..

[18] Asfar S., Abdeen S., Dashti H., Khoursheed M., Al-Sayer H., Mathew T., Al-Bader A. (2003). Effect of green tea in the prevention and reversal of fasting-induced intestinal mucosal damage. Nutrition.

[19] Yi R., Wang R., Sun P., Zhao X. (2015). Antioxidant-mediated preventative effect of Dragon-pearl tea crude polyphenol extract on reserpine-induced gastric ulcers. Exp. Ther. Med..

[20] Kuo K.-L., Weng M.-S., Chiang C.-T., Tsai Y.-J., Lin-Shiau S.-Y., Lin J.-K. (2005). Comparative Studies on the Hypolipidemic and Growth Suppressive Effects of Oolong, Black, Pu-erh, and Green Tea Leaves in Rats. J. Agric. Chem..

[21] Wong J.Y., Abdulla M.A., Raman J., Phan C.W., Kuppusamy U.R., Golbabapour S., Sabaratnam V. (2013). Gastroprotective effects of Lion’s mane mushroom *Hericium erinaceus* (Bull.:Fr.) Pers. (Aphyllophoromycetideae) extract against ethanol-induced ulcer in rats. Evid. Based Complement. Alternat. Med..

[22] Pillai S., Oresajo C., Hayward J. (2005). Ultraviolet radiation and skin aging: Roles of reactive oxygen species, inflammation and protease activation, and strategies for prevention of inflammation-induced matrix degradation—A review. Int. J. Cosmet. Sci..

[23] Del Pino-García R., González-Sanjosé M.L., Rivero-Pérez M.D., García-Lomillo J., Muñiz P. (2016). Total antioxidant capacity of new natural powdered seasonings after gastrointestinal and colonic digestion. Food Chem..

[24] Bonthius D.J., Winters Z., Karacay B., Bousquet S.L., Bonthius D.J. (2015). Importance of genetics in fetal alcohol effects: Null mutation of the *nNOS* gene worsens alcohol-induced cerebellar neuronal losses and behavioral deficits. Neurotoxicology.

[25] Lee M., Hyun D., Jenner P., Halliwell B. (2001). Effect of proteasome inhibition on cellular oxidative damage, antioxidant defences and nitric oxide production. J. Neurochem..

[26] Kosenko E.A., Tikhonova L.A., Alilova G.A., Montoliu C., Barreto G.E., Aliev G., Kaminsky Y.G. (2017). Portacaval shunting causes differential mitochondrial superoxide production in brain regions. Free. Radic. Boil. Med..

[27] Suo H., Zhao X., Qian Y., Sun P., Zhu K., Li J., Sun B. (2016). *Lactobacillus fermentum* Suo Attenuates HCl/Ethanol Induced Gastric Injury in Mice through Its Antioxidant Effects. Nutrients.

[28] Li N.S., Luo X.J., Zhang Y.S., He L., Liu Y.Z., Peng J. (2011). Phloroglucinol protects gastric mucosa against ethanol-induced injury through regulating myeloperoxidase and catalase activities. Fundam Clin. Pharmacol..

[29] Liu H.B., Huang X.D., Liu M.H., Shanguan J.Y. (2002). Significance of NOS expressions in gastric carcinoma. Med. J. Nat. Defend. For. Northwest China.

[30] Ignarro L.J., Byrns R.E., Sumi D., De Nigris F., Napoli C. (2006). Pomegranate juice protects nitric oxide against oxidative destruction and enhances the biological actions of nitric oxide. Comp. Toxicogenomics.

[31] Mahmoud Y.I., El-Ghffar E.A.A. (2019). Spirulina ameliorates aspirin-induced gastric ulcer in albino mice by alleviating oxidative stress and inflammation. Biomed. Pharmacother..

